# High-grade astroblastoma initially misdiagnosed as papillary meningioma: a case report

**DOI:** 10.11604/pamj.2025.51.99.48550

**Published:** 2025-08-19

**Authors:** Anass Haloui, El Farissi Mohamed Amine, Daoudi Chaimae, Dahamou Mohamed, Amine Kada, Khay Hamid, Khoulali Mohammed, Benheddi Maryam, Hicham Benramdane, Imane Kamaoui, Imane Skiker, Nassira Karich, Amal Bennani

**Affiliations:** 1 Laboratory of Pathological Anatomy, Mohammed VI University Hospital, Faculty of Medicine and Pharmacy of Oujda, Mohammed First University, Oujda, Morocco,; 2 Department of Neurosurgery, Mohammed VI University Hospital, Faculty of Medicine and Pharmacy of Oujda, Mohammed First University, Oujda, Morocco,; 3 Department of Radiology, Mohammed VI University Hospital, Faculty of Medicine and Pharmacy of Oujda, Mohammed First University, Oujda, Morocco

**Keywords:** Astroblastoma, papillary meningioma, pseudorosettes, central nervous system, case report

## Abstract

Astroblastoma is a well-circumscribed glial neoplasm that typically occurs in the cerebral hemisphere of children and young adults, usually presenting with symptoms of increased intracranial pressure such as headaches, vomiting, and seizures. It is characterized histologically by papillary or pseudopapillary architecture, astroblastic pseudorosettes, vascular hyalinization, and lack of fibrillarity. However, these morphological features are not considered pathognomonic to astroblastomas and may be encountered in several central nervous system tumors, such as ependymoma and papillary meningioma, complicating the diagnostic process. In this report, we describe the case of an astroblastoma in an 18-year-old male presenting with symptoms of intracranial hypertension. Imagery revealed a right cerebral hemispheric mass with a particularly misleading fronto-ethmoidal dural attachment, which, in conjunction with the deceptive papillary architecture, prompted the initial misdiagnosis of papillary meningioma on two consecutive occasions before diagnostic revision. This article aims to highlight the main reasons for this diagnostic confusion, along with providing key clinical, radiological, histological, and molecular features of relevance to the differential diagnostic approach.

## Introduction

Astroblastoma is characterized by papillary or pseudopapillary architecture, astroblastic pseudorosettes, perivascular hyalinization, and lack of fibrillarity [1]. It mainly occurs in the cerebral hemispheres of children and young adults. Despite being rare, astroblastoma should be considered in the differential diagnosis of well-circumscribed supratentorial masses in young patients, who often present with headaches, nausea, vomiting, and seizures, especially when pseudorosettes or papillary architecture are present. The pseudorosettes formations can be misleading, as they can also be encountered in various central nervous tumors that may affect the cerebral hemispheres, such as ependymomas and other papillary neoplasms. Moreover, atypical anatomical localizations of astroblastoma within the central nervous system may further complicate the diagnosis. Illustratively, in the present case, a right cerebral hemispheric mass in an 18-year-old male with no pathological history was initially misdiagnosed as a papillary meningioma on two separate occasions due to its dural attachment and radiological resemblance to meningioma, before diagnostic revision after recurrence of the tumor. Therefore, thorough knowledge of the clinical context and imagery features, along with careful morphological analysis, is mandatory to avoid misdiagnosis.

## Patient and observation

**Patient information:** an 18-year-old man with no notable pathological history presented with symptoms of intracranial hypertension, including increasing headaches of 4 months' duration, nausea, and progressively diminishing vision of the right eye.

**Clinical findings:** neurological examination revealed a positive Barre and Mingazzini sign limited to the left side, and a grade 1 exophthalmos of the right eye.

**Figure 1:**
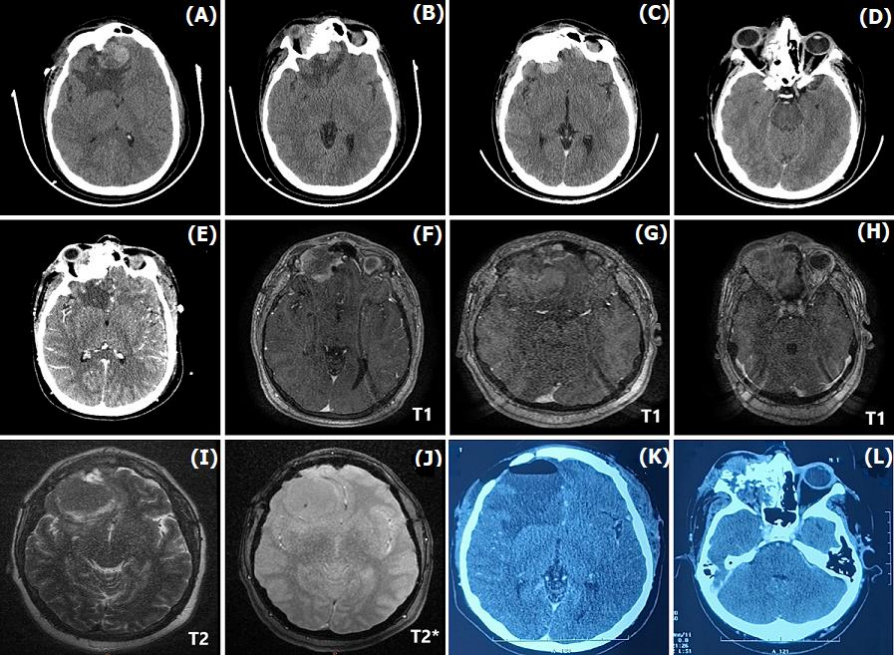
A, B, C, D, E, F, G, H, I, J, K, L) evolution of a right frontal extra-axial mass with recurrences and extensions, confirmed by computed tomography and magnetic resonance imaging

**Timeline of current episode:** in April 2024, the initial cerebral CT scan performed revealed a well-circumscribed right paramedian basifrontal extra-axial mass, measuring 37 x 30 mm, spontaneously hyperdense and heterogeneous, with fronto-ethmoidal dural attachment, initially raising suspicion of a meningioma. It exerts a mild mass effect on the right lateral ventricle and extends into the right orbit, causing grade 1 exophthalmos (Figure 1A, Figure 1B). The patient underwent an initial surgical resection of the cerebral portion of the tumor, whereas the orbital portion was left untouched (Figure 1C, Figure 1D). Two months later, in June 2024, the tumor recurred at the same location, and the patient underwent a second surgical resection. Postoperative CT and MRI demonstrated complete resection of the cerebral portion of the tumor, along with persistence of the right orbital extension (Figure 1E, Figure 1F). In February 2025, the patient returned with the same symptomatology along with an aggravated grade 3 exophthalmos of the right eye. A new cerebral MRI was performed, showing a right hemispheric extra-axial mass measuring 48 x 47 mm, with a clear pushing border, a moderate mass effect with partial compression of the ipsilateral ventricle, and an extension into the right ethmoidal sinus and orbital cavity (Figure 1G, Figure 1H, Figure 1I, Figure 1J). The patient underwent a third complete surgical resection of the brain tumor before being referred to the radiotherapy department for adjuvant radiotherapy (Figure 1K, Figure 1L). Table 1 resumes the timeline of the patient's history.

**Table 1:** timeline of current episode

April 2024	June 2024	February 2025
The Patient presented with symptoms of intracranial hypertension (increasing headaches, nausea, and progressively diminishing vision)	First tumoral recurrence at the same location, measuring 18 × 17 mm, with progression of endo-orbital extension causing grade 2 exophthalmos. (Figure 1: C, D)	Second recurrence with aggravated exophthalmos of the right eye.
Cerebral CT and MRI revealed: Right basifrontal extra-axial mass, measuring 37 × 30 mm, with fronto-ethmoidal attachment and grade 1 exophthalmos, initially raising suspicion of a meningioma. (Figure 1: A, B)	Second surgical resection	A new cerebral MRI: right hemispheric extra-axial mass, with clear pushing border and a moderate mass effect with partial compression of the ipsilateral ventricle measuring 48x47 mm, with extension into the right ethmoidal sinus and orbital cavity (Figure 1: G, H, I, J)
The Patient underwent the first surgical resection of the intracranial portion of the tumor	Postoperative MRI demonstrated complete resection of the cerebral tumor, along with persistence of the right orbital extension (Figure 1: E, F)	Third complete surgical resection of the brain tumor (Figure 1: K, L)
Initial diagnosis: papillary meningioma of grade 3	-	Diagnosis reconsideration: astroblastoma
-	-	Patient referred to the radiotherapy department for adjuvant radiotherapy

**Figure 2:**
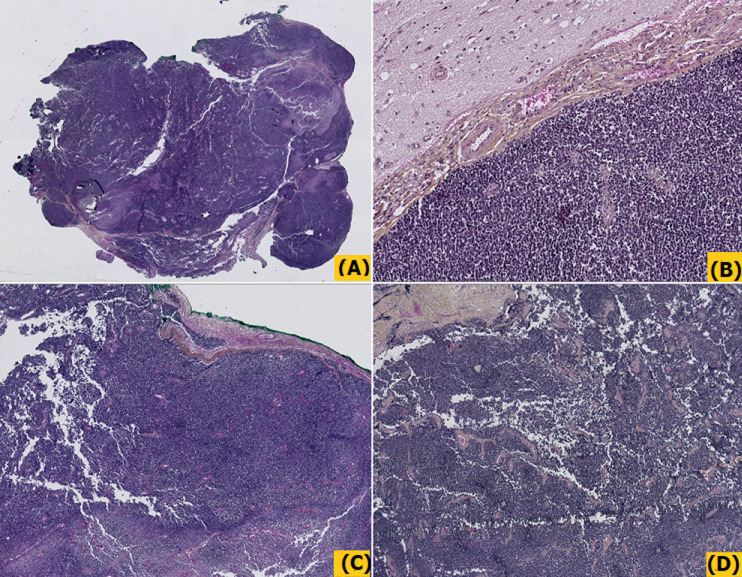
A, B) photomicrographs from two consecutive resections showing the well-circumscribed borders of the tumor, with pushing borders facing adjacent normal cerebral parenchyma; C, D) the overall architecture was marked by solid sheets with frequent emergence of pseudopapillary and papillary patterns, (Hematoxylin, Eosin and Safran stain, Magnification x2,5, x5, and x10)

**Figure 3:**
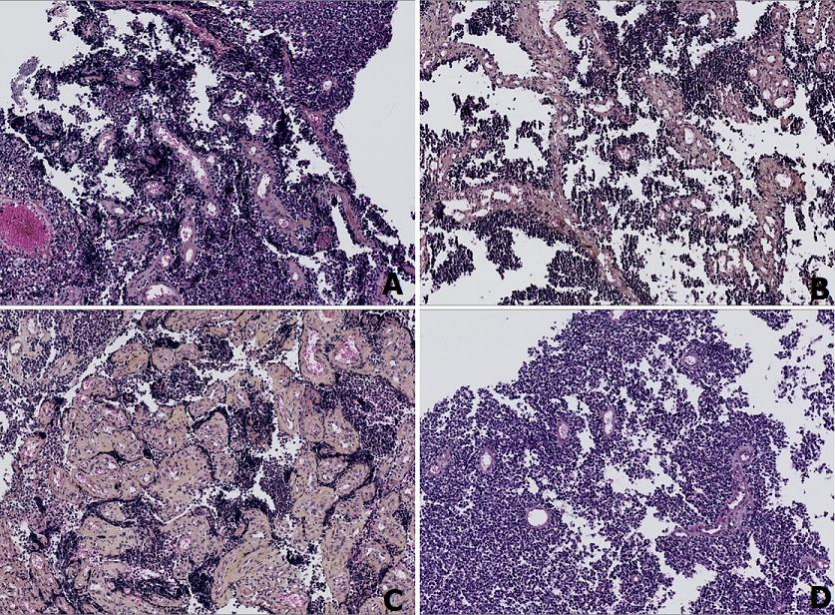
A, B, C, D) photomicrographs showing the various appearances of pseudorosettes depending on the plane of section, transverse sections highlight the perivascular arrangement, while longitudinal sections reveal the radial orientation of tumor cells around blood vessels (Hematoxylin, Eosin, and Safran stain, Magnification x10)

**Figure 4:**
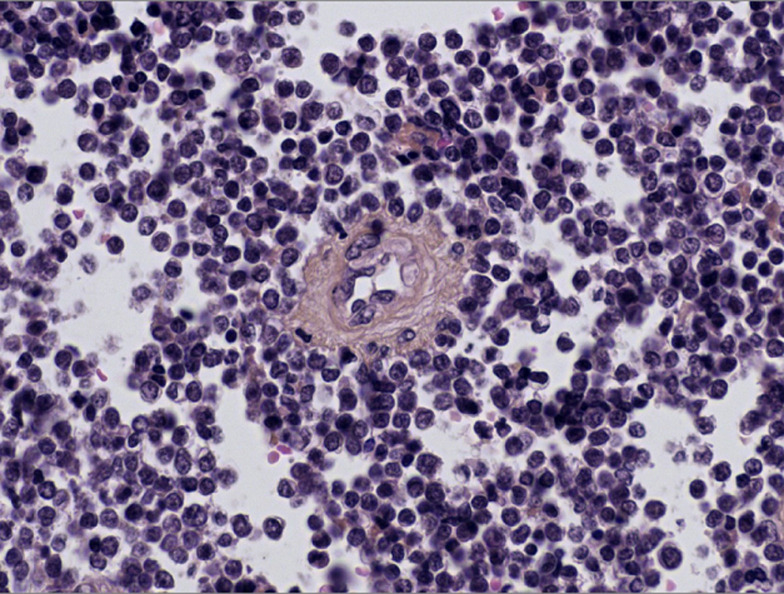
high power view showing an 'astroblastic' pseudorosette, characterized by tumor cells radially anchored to a central blood vessel, the cytoplasmic processes are stout, shorter, and wider than those found in ependymoma (Hematoxylin, Eosin, and Safran stain, Magnification x40)

**Figure 5:**
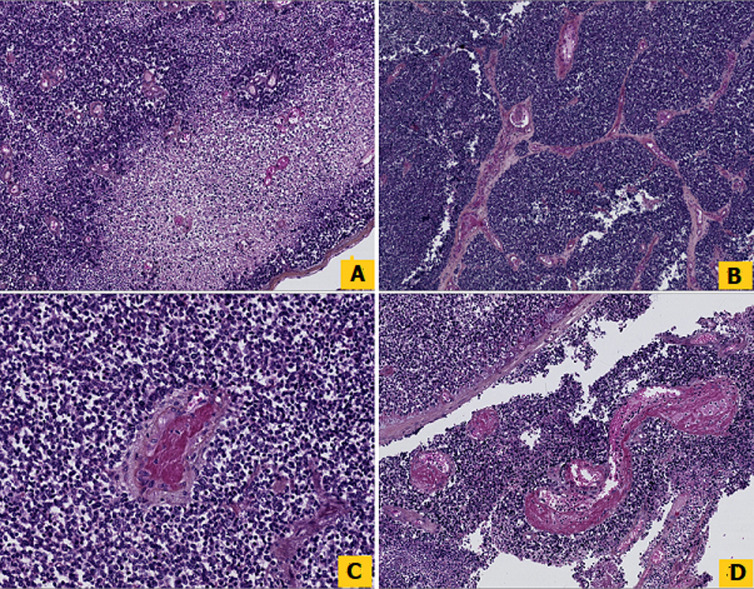
A) foci of tumoral necrosis; B) rich vascular network set in the stroma; C, D) lack of fibrillarity both in the background and within pseudorosettes, with frequent perivascular hyalinization (Hematoxylin, Eosin, and Safran stain, Magnification x10 and x20)

**Figure 6:**
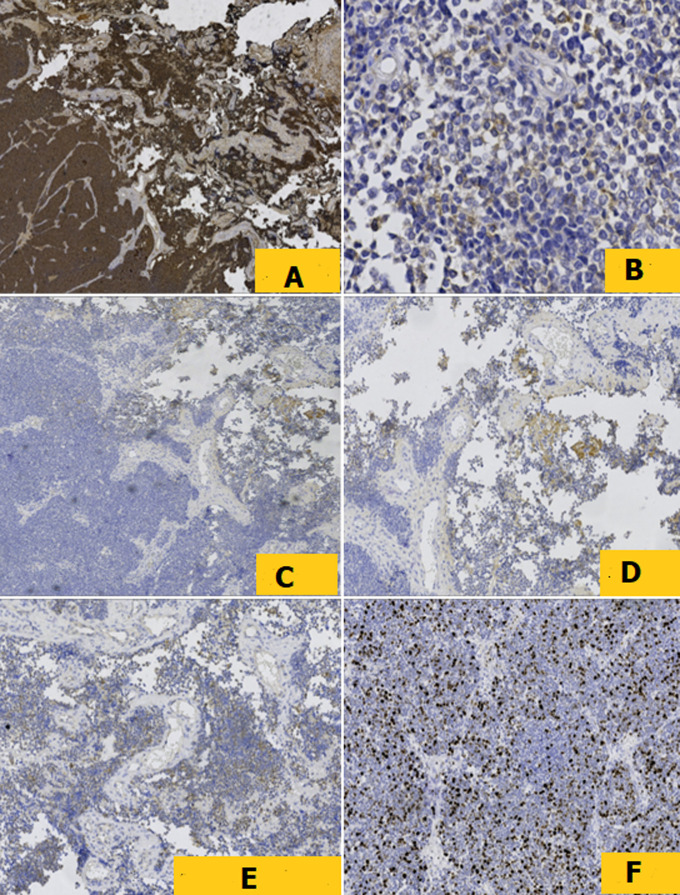
A) immunohistochemistry revealed diffuse expression of PS100, B) patchy and weak expression of EMA and C) synaptophysin, D, E) mainly negative GFAP with some rare positive cells, F) and Ki67 estimated at 60

**Diagnostic assessment:** histopathological examination of all three subsequent resection specimens revealed consistent features. The well-circumscribed nature of the tumor with pushing margins was not identified until the final resection specimen, in which a small portion of adjacent normal cerebral parenchyma was sampled along with the tumor (Figure 2A, Figure 2B). The overall architecture was marked by solid sheets (Figure 2C, Figure 2D), with focal emergence of pseudorosettes characterized by tumor cells radially anchored to a central blood vessel by stout, short, and wide cytoplasmic processes, conferring a papillary or pseudopapillary pattern to the tumor, initially raising suspicion of a papillary meningioma (Figure 3). The tumor cells were cuboidal, round, or elongated, provided with a medium-sized nucleus with dense or slightly vesicular chromatin, occasional conspicuous nucleoli, and abundant eosinophilic or clear cytoplasm (Figure 4). Numerous mitotic figures and tumor necrosis were observed (Figure 5A). The tumor stroma lacked fibrillarity and contained a rich vascular network of thickened-wall vessels with frequent perivascular hyalinization (Figure 5B, Figure 5C, Figure 5D). Immunohistochemical examination revealed diffuse expression of S100, patchy and weak expression of Epithelial membrane antigen (EMA) and synaptophysin, an overall negativity of glial fibrillary acidic protein (GFAP) with only rare positive cells, and absence of immunoreactivity for chromogranin, CD56, and OLIG2. The mitotic index was elevated, with Ki67 estimated at 60%. Neurofilament did not reveal any entrapped neurons within the tumor, supporting the solid pattern of the tumor with pushing borders (Figure 6). The initial diagnosis was World Health Organization (WHO) grade 3 papillary meningioma. But given the unusual clinical course and poor response to treatment, a revision of the diagnosis integrating morphology, immunohistochemistry, imagery, and clinical context concluded to a high-grade astroblastoma.

**Diagnosis:** the final diagnosis is high-grade astroblastoma. The high grade was granted, given the brisk mitotic activity, elevated KI67, nuclear atypia, and tumoral necrosis foci.

**Therapeutic interventions:** the patient underwent three subsequent surgical resections of the tumor followed by radiotherapy.

**Follow-up and outcome of interventions:** to date, the patient remains in good clinical condition, with no reported complications.

**Patient perspective:** the patient feels more relieved after the last surgery and radiotherapy.

**Informed consent:** the patient gave informed consent.

## Discussion

As originally described by Bailey *et al*. in 1926 [2], astroblastoma was defined based on the finding of astroblastic perivascular pseudorosettes within a glial neoplasm. More recently, the definition has been refined to emphasize a tumor composed entirely of astroblastic elements, along with circumscription, vascular hyalinization, and lack of fibrillarity [1]. It affects children and young adults, with a median age of 15, and usually occurs in the cerebral hemispheres, although intraventricular and brainstem localization were rarely described. On imagery, astroblastomas appear as well-demarcated, solid or cystic masses that are isointense on T1 and hyperintense on T2 [3]. Acquired fusion MN1-BEND2 is currently considered the key driver of the tumor's oncogenic pathway [3].

On a morphological level, a pattern-based approach is greatly useful in narrowing the differential diagnosis when confronted with a supratentorial tumor displaying papillary or pseudopapillary configuration. Multiple tumors may display similar architectural features, closely mimicking astroblastoma. Among these tumors, papillary meningioma can be particularly misleading and challenging to rule out, as illustrated in our case, in which astroblastoma was mistaken for a papillary meningioma on two separate occasions. Upon diagnosis revision, several reasons that accounted for the initial diagnostic confusion are discussed. The first reason is the location of the tumor. In contrast to astroblastoma, papillary meningioma is more often dural-based than intraparenchymal. Therefore, in this particular case, the dural attachment of the tumor seen in imagery and intraoperatively confused the diagnostic approach. The second reason is morphological and specimen-related. Papillary meningioma usually contains, in addition to the distinct papillary architecture, areas of well-known histologic variants of meningioma, such as meningothelial whorls [4]. However, the biopsy nature of the specimen led us to assume that areas more suggestive of meningioma may simply not have been included in the submitted tissue. One peculiar feature that should have prompted further consideration, which we unfortunately overlooked and proved to be decisive in the current case, was the sharp demarcation from the adjacent brain tissue with the characteristic pushing border. Indeed, papillary meningioma may be brain invasive in contrast to the sharp demarcation of astroblastoma [5]. In a series of 17 papillary meningioma reported by Ludwin *et al*., 47% of cases showed histological signs of brain invasion [6]. Nevertheless, the sharp demarcation was not identified in our case until the last surgical resection, highlighting the importance of thorough sampling of the specimen. In the same series of Ludwin *et al*. 59% of cases developed one or more local recurrences [6]. Therefore, at the time of the first recurrence in our case, the diagnosis of papillary meningioma was not ruled out yet because of the well-known propensity of this subtype to invade both bone and cerebral parenchyma, to recur locally and even to metastasize in extracranial sites [7]. It wasn't until the second recurrence with the unusual clinical behavior and non-response to surgical management that further review of the initial diagnosis was performed, resulting in an astroblastoma.

An additional reason contributing to the misdiagnosis was the deceptive immunohistochemical profile in the present case, especially the overall negativity of GFAP and the focal expression of EMA. While patchy expression of EMA is highly suggestive of meningioma, focal EMA staining, sometimes with a dot-like pattern, can also be encountered in astroblastoma [8]. Furthermore, Ki67 is usually elevated in both high-grade astroblastomas and WHO grade 3 meningiomas; therefore, segregation between the two entities cannot be reliably done on the basis of proliferation index. Even though the lack of GFAP expression is possible in astroblastomas, it was more consistent at the time with the diagnosis of papillary meningioma, since the latter, as a rule, lacks GFAP positivity. GFAP expression in astroblastoma is variable depending on the molecular subtype. Lehman and al. performed RNA sequencing of 24 supratentorial astroblastoma-like tumors, revealing several molecular subgroups [9]. Tumors harboring MN1-BEND2 fusion formed a distinct cluster, demonstrating high expression of ependymal genes (example: SOX14, RFX3, YAP1) and low expression of glial (example: OLIG2, GFAP) and neuronal genes (example: GRIA2, MAOA), with absent or patchy immunoreactivity to GFAP, supporting the assumption that these tumors more likely derive from early ventricular radial glia ependymal progenitors. In contrast, another distinct cluster, referred to as the mitogen-activated protein kinase (MAPK) supercluster, comprised tumors characterized by mutation of MAPK pathway genes and/ or PI3K/AKT/mTOR pathway. These tumors demonstrated high expression of genes reflecting glial and neural differentiation, and showed diffuse immunoreactivity to GFAP, further supporting the hypothesis that they may originate from Outer Radial Glia or Truncated Radial Glial cells [9]. In our case, the negativity of GFAP suggests that the tumor may harbor MN1-BEND2 fusion. Unfortunately, molecular profiling was not conducted, limiting the ability to confirm the tumor's placement within one of the defined molecular subgroups.

Several other glial and glioneuronal tumors may also share deceptively similar morphological features to astroblastoma. Table 2 summarizes the key clinical, morphological, and immunohistochemical features of tumors that may mimic astroblastoma in the central nervous system. Accurate diagnosis requires integration of histopathology, immuno-molecular profile, and radiological context.

**Table 2:** key clinical, morphological, and immunohistochemical features of differential diagnoses of astroblastoma

Tumor	Age/location	Morphology	Immunohistochemistry
Astroblastoma	Children and young adults (3 months to 40 years, median 15 years). Essentially intra-axial: (Cerebral hemispheres) Secondary attachment to dura possible + (extra-axial localization)	Sharp demarcation with pushing borders. Astroblastic pseudorosettes (stout processes + shorter and wider than ependymomas). Lack of fibrillarity. Perivascular Hyalinization. High grade: hypercellularity, Atypia, Mitosis > 5/10HPF, Microvascular Proliferation, Necrosis	GFAP variable; S100 Variable; EMA focally +; CK - Synaptophysin-Neurofilament - Ki-67 is elevated in high-grade
Supratentorial Ependymoma (papillary variant)	Children and young adults. Around or within the ventricular system (Lateral ventricle +).	Sharp demarcation. True ependymal rosettes. Perivascular pseudorosettes. Fibrillary background	GFAP+, EMA+ (dot-like) S100+; Olig2− ; CK - (rarely focally +), Neurofilament-
Papillary meningioma	Adults, rare in children (median age 66 years) Cerebrospinal axis (Intracranially: over the convexity and parasagittal regions) Dural attachment	Brain invasion frequent + Papillary or pseudopapillary pattern Areas of classic meningioma (thorough sampling +) Vascular hyalinization and thickening the possible. Mitotic activity and Necrosis are possible	GFAP - ; EMA patchy ; PS100+; CK- or only focally+
Papillary tumor of pineal region (PTPR)	Children and young adults (median 35 years) Pineal region	Well-demarcated, Papillary and solid areas. Columnar cells, round or oval nuclei, pale to eosinophilic cytoplasm. Focal necrosis, Vascular hyalinization	CK+, S100+, NSE+ Vimentin+, GFAP variable, EMA variable, Olig2−; CD56+ Synaptophysin variable Chromogranin variable
Papillary glioneuronal tumor (PGNT)	Young adults (mean age of 27) Cerebral hemispheres (temporal lobe+)	Well circumscribed, solid or cystic. Biphasic: Pseudopapillary glial structures + Interpapillary neuronal cells	GFAP + in glial component Synaptophysin + in neuronal component
Angiocentric glioma	Children and young adults (mean age 17) Cerebral hemispheres are centered in the cortex and often extends into subcortical regions	Infiltrative growth pattern Uniform bipolar cells, spindle-shaped Perivascular distribution: parallel to vessels, sometimes radially (reminiscent of pseudorosettes) Subpial distribution (palisading appearance). Mitosis usually absent	GFAP +; PS100 +; CK-; EMA+ dot like Neurofilament + in entrapped neurons, supporting the infiltrative pattern
Choroid plexus carcinoma	Children, usually < 3 years Lateral ventricle +	True papillae, Solid areas possible Nuclear atypia, Necrosis, mitosis	CK+; CK7+; CK20-; PS100+ Transthyretin+; GFAP-; EMA−, Vimentin+
High-grade diffuse glioma (epithelioid type)	Any age, usually adults Cerebral hemispheres (also affect the brainstem, cerebellum, and Spinal cord)	Ill-defined, infiltrative Subpial condensation Perivascular Aggregates. Perineuronal satellitosis Hypercellularity, Nuclear atypia; Necrosis; Microvascular proliferation	GFAP+, Olig2+, S100+, variable EMA+, CK−

## Conclusion

The diagnosis of astroblastoma can be very challenging, given the morphological similarities with other cerebral tumors. This case emphasizes the diagnostic pitfalls associated with astroblastoma, particularly in atypical presentations, along with providing an overview of the clinical, radiological, histological, and molecular findings essential to its accurate identification and differentiation from mimickers.
